# Chronic esophagotracheal fistula secondary to esophageal diverticulum successfully treated by endoscopic submucosal dissection and dual action tissue clip

**DOI:** 10.1055/a-2163-2050

**Published:** 2023-10-24

**Authors:** Lei Shi, Fei Long, Hongwei Xu, Na Chen, Jian Ge, Ruzhen Jia, Junmei Jiang

**Affiliations:** 1 Department of Gastroenterology, Shandong Provincial Hospital Affiliated to Shandong First Medical University, Jinan, China; 2 Department of Gastroenterology, The Third Affiliated Hospital of Shandong First Medical University, Jinan, China

Esophagotracheal fistula secondary to esophageal diverticulum is rare but challenging to treat 1 . Some cases of esophagotracheal fistula successfully treated by endoscopic submucosal dissection (ESD) have been reported 2 3 4 , but to our knowledge we report the first case of esophagotracheal fistula secondary to esophageal diverticulum successfully treated by ESD and clip closure.

We present the case of a 64-year-old man who contracted recurrent pulmonary infections over 2 years. The upper digestive tract showed a niche formation in the right wall of the esophagus at the T7 vertebral body level, but no obvious leakage of contrast agent was found ( Fig. 1 a ). Computed tomography (CT) scan showed inflammation in the right lower lobe, with partial atelectasis ( Fig. 2 a ). Gastroscopy revealed a diverticulum in the right lateral wall of the esophagus and a 5-mm fistulous orifice inside the diverticulum ( Fig. 3 a ). After anti-infective treatment for 3 days, we performed ESD for the esophagotracheal fistula and esophageal diverticulum ( Video 1 ).

**Fig. 1:**
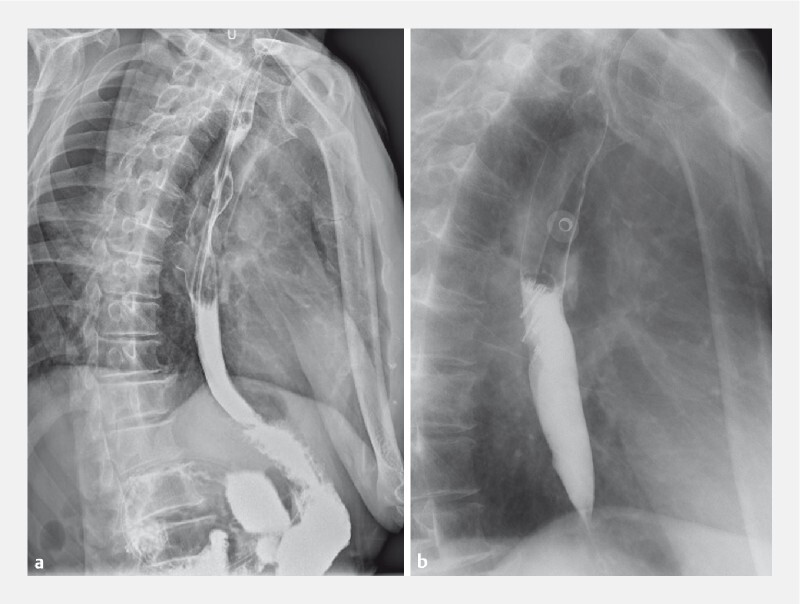
Radiographic images. **a** A niche formation in the right wall of the esophagus at the T7 vertebral body level. **b** Resolution of the esophageal diverticulum.

**Fig. 2:**
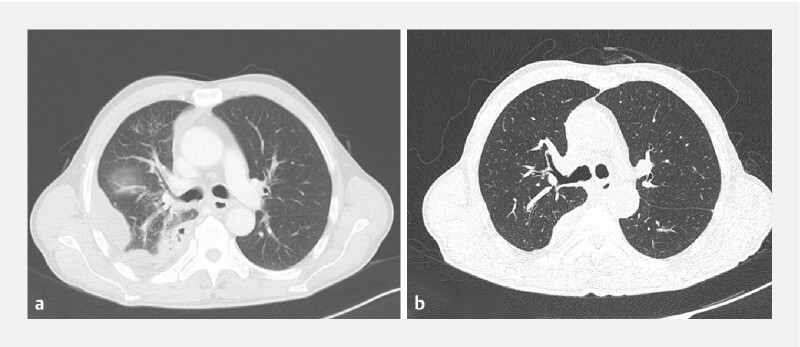
Computed tomography scan. **a** Inflammation in the right lower lobe with partial atelectasis. **b** The pulmonary inflammation was significantly resolved.

**Fig. 3:**
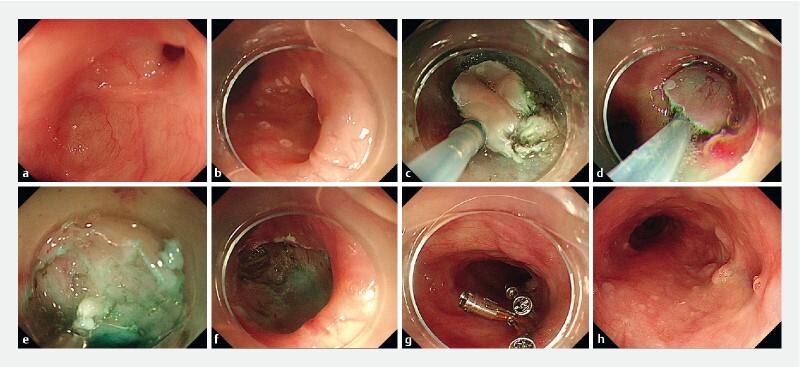
Endoscopic images showing the endoscopic submucosal dissection procedure. **a** A diverticulum in the right lateral wall of the esophagus and a fistulous orifice inside the diverticulum. **b** Marking the surrounding mucosa of the esophageal diverticulum. **c** Dissecting the mucosal and submucosal layers inside the diverticulum. **d** Excision of the mucosal and submucosal layers with a snare trap. **e** Dissecting the mucosal patch surrounding the fistulous orifice. **f** Cutting off part of the muscularis propria inside the diverticulum. **g** Closure of the exposed area with Dual Action Tissue clips and SureClips (Micro-Tech Endoscopy, USA Inc., Ann Arbor, Michigan, USA). **h** Gastroscopy showed that the esophagotracheal fistula was healed after 3 months.

**Video 1** The endoscopic submucosal dissection procedure was performed to dissect the mucosal and submucosal layers inside the diverticulum, followed by clip closure.


After marking the surrounding mucosa of the esophageal diverticulum and injecting the submucosal layer, we dissected the mucosal and submucosal layers inside the diverticulum ( Fig. 3 b, c, d ). Then, a coagulation forceps was used to dissect the mucosal patch surrounding the fistulous orifice to increase the chances of successful scarring ( Fig. 3 e ). Furthermore, we resected part of the muscularis propria inside the diverticulum to prevent incomplete closure of the diverticulum ( Fig. 3 f ). Finally, two Dual Action Tissue clips (Micro-Tech Endoscopy, USA Inc., Ann Arbor, Michigan, USA) and three SureClips (Micro-Tech Endoscopy, USA Inc.) were used to close the exposed area ( Fig. 3 g , Video 1 ). The patient successfully restarted oral food intake and was discharged.

Repeat upper gastrointestinal radiography showed resolution of the esophageal diverticulum ( Fig. 1 b ), and CT scan showed resolution of the pulmonary inflammation ( Fig. 2 b ). Repeat gastroscopy confirmed the closure of the orifice during the subsequent 3 months ( Fig. 3 h ).

This case demonstrates that ESD and diverticulum closure by clips can be a valuable procedure for treating chronic esophagotracheal fistula secondary to esophageal diverticulum.

Endoscopy_UCTN_Code_CCL_1AB_2AC_3AF

## Notes

Endoscopy E-Videos

https://eref.thieme.de/e-videos
*E-Videos* is an open access online section of the journal *Endoscopy* , reporting on interesting cases and new techniques in gastroenterological endoscopy. All papers include a high-quality video and are published with a Creative Commons CC-BY license. Endoscopy E-Videos qualify for HINARI discounts and waivers and eligibility is automatically checked during the submission process. We grant 100% waivers to articles whose corresponding authors are based in Group A countries and 50% waivers to those who are based in Group B countries as classified by Research4Life (see: https://www.research4life.org/access/eligibility/ ). This section has its own submission website at https://mc.manuscriptcentral.com/e-videos
